# Biology and Pathogenesis of SARS-CoV-2: Understandings for Therapeutic Developments against COVID-19

**DOI:** 10.3390/pathogens10091218

**Published:** 2021-09-19

**Authors:** Homa Nath Sharma, Charity O. D. Latimore, Qiana L. Matthews

**Affiliations:** 1 Microbiology Program, Department of Biological Sciences, Alabama State University, Montgomery, AL 36104, USA; hsharma1952@myasu.alasu.edu; 2 Department of Biological Sciences, Alabama State University, Montgomery, AL 36104, USA; Clatimore3784@myasu.alasu.edu

**Keywords:** coronaviruses, HCoVs, SARS-CoV-2, COVID-19, host-virus interaction, immune-evasion, pathogenesis, animal models, vaccines, antivirals, EVs

## Abstract

Coronaviruses are positive sense, single-stranded, enveloped, and non-segmented RNA viruses that belong to the Coronaviridae family within the order Nidovirales and suborder Coronavirinae. Two Alphacoronavirus strains: HCoV-229E and HCoV-NL63 and five Betacoronaviruses: HCoV-HKU1, HCoV-OC43, SARS-CoV, MERS-CoV, and SARS-CoV-2 have so far been recognized as Human Coronaviruses (HCoVs). Coronavirus disease 2019 (COVID-19) caused by SARS-CoV-2 is currently the greatest concern for humanity. Despite the overflow of research on SARS-CoV-2 and other HCoVs published every week, existing knowledge in this area is insufficient for the complete understanding of the viruses and the diseases caused by them. This review is based on the analysis of 210 published works, and it attempts to cover the basic biology of coronaviruses, including the genetic characteristics, life cycle, and host-pathogen interaction, pathogenesis, the antiviral drugs, and vaccines against HCoVs, especially focusing on SARS-CoV-2. Furthermore, we will briefly discuss the potential link between extracellular vesicles (EVs) and SARS-CoV-2/COVID-19 pathophysiology.

## 1. Introduction

**Figure 1:**
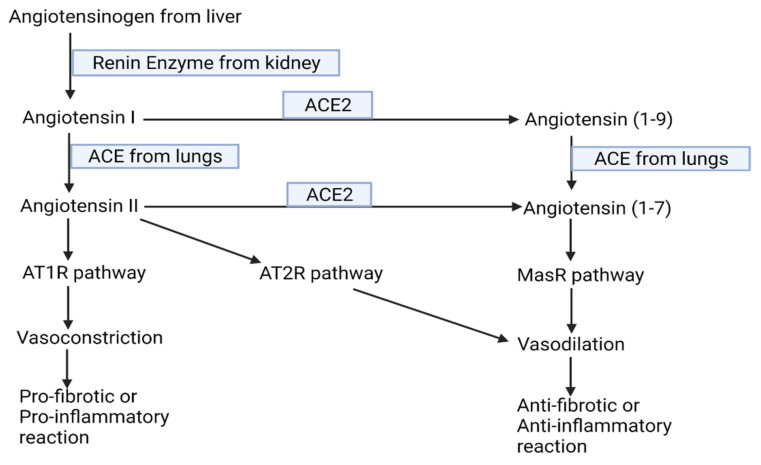
Flowchart of RAS. ACE, Angiotensin converting enzyme; AT1R, Angiotensin type 1 receptor; AT2R, type 2 receptor; MasR, Mas receptor. The boxes wrap the relevant enzymes catalyzing the reaction (created with BioRendor.com. Accessed on 9 September 2021).

HCoVs have long been known to spread in the world population. Severe acute respiratory syndrome-coronavirus (SARS-CoV), severe acute respiratory syndrome-coronavirus-2 (SARS-CoV-2), and Middle East respiratory syndrome-coronavirus (MERS-CoV) have appeared most recently within the last 20 years in the human population. SARS-CoV, SARS-CoV-2, and MERS-CoV are relatively highly pathogenic as compared to other endemic species of HCoVs [1,2]. COVID-19, which is caused by SARS-CoV-2, initially appeared in China in December 2019, has now circulated worldwide, and been declared as a pandemic [3,4]. As of 2 September 2021, a total of 218,205,951 cases of COVID-19, including 4,526,583 deaths, as per the data released from World Health Organization (WHO) have been confirmed globally (https://covid19.who.int/. Accessed on 2 September 2021). About 10–15% of all COVID-19 cases have been reported to develop severe disease and approximately 5% shows critical illnesses [5]. On the basis of analysis of whole-genome sequencing (WGS) and whole -exome sequencing (WES) data on host individuals, critical illnesses can be correlated with genes involved in innate antiviral defense such as Interferon Alpha and Beta Receptor Subunit 2 (IFNAR2) and Oligoadenylate Synthetase (OAS), and the genes mediating the life-threatening late phage of COVID-19 such as Dipeptidyl Peptidase 9 (DPP9), Tyrosine Kinase 2 (TYK2), and Chemokine (C-C motif) Ligand 2 (CCL2). The genetics of individuals has thus been attributed to the susceptibility and host response elicited by SARS-CoV-2 as well as the severity of COVID-19 disease [6,7]. On the other hand, blocking of ADAM-17 mediated Angiotensin converting enzyme 2 (ACE2) shedding and upregulation of ACE2-Angiotensin-1-7-MasR axis of Renin Angiotensin System (RAS) (see Figure 1 for detailed information regarding this pathway) have been found to mediate enhanced COVID-19 severity in males [8]. Similarly, a mutation in the Toll-Like Receptor (TLR) 7 gene has also been suggested as another underlining mechanism for higher susceptibility of males towards COVID-19 [9]. Despite the overflow of research on SARS-CoV-2 and other HCoVs published every week, existing knowledge in this area is insufficient for complete understanding of viruses and the disease caused by them. So only the exhaustive studies on the virology of SARS-CoV-2, genetics of host response and pathophysiology can offer the basis for the development and advancement of preventive as well as therapeutic strategies against the disease [10].

## 2. Coronaviruses

**Figure 2:**
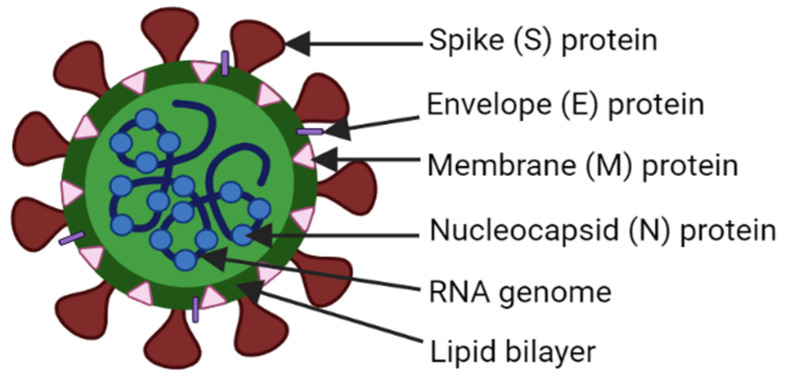
A typical virion structure of a coronavirus (created with BioRender.com. Accessed on 28 July 2021).

Coronaviruses are positive sense, single-stranded, enveloped RNA viruses that belong to the Coronaviridae family within the order Nidovirales and suborder Coronavirinae [1,11,12]. Expression of the replicase polyprotein via ribosomal frameshifting, gene expression from 3′-nested subgenomic RNAs (sg RNAs) and unique activities of enzymes in the replicase protein products are the peculiar features of not only coronaviruses, but all Nidoviruses [13]; including toroviruses, roniviruses, and arteriviruses, in addition to the coronaviruses [13,14]. Coronaviruses are 80–120 nm in diameter, with extreme sizes approximately as small as 50 nm and as large as 200 nm: roughly spherical and moderately pleomorphic. The non-segmented genome has a length ranging from 27.3 to 31.1 kb, making this the largest among RNA genomes. As in eukaryotes, their messenger RNA (mRNA) also have 5′-cap and 3′-polyA tails [2,13]. The general structure of coronavirus has been depicted in the Figure 2 below.

**Figure 3:**
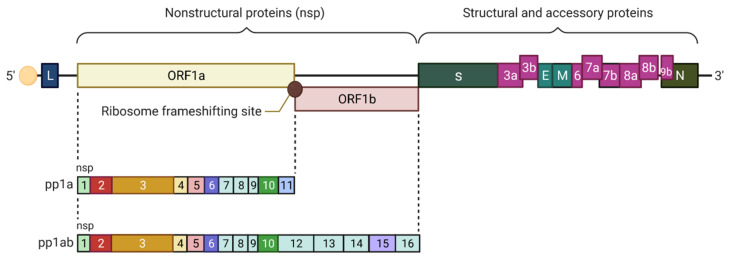
Genomic organization of SARS-CoV, where one third of the right part of the genome encodes nine accessory proteins (3a, 3b, 6, 7a, 7b, 8a, 8b and 9b). These are interspersed between genes encoding structural proteins (S, E, M, N). UTRs are flanking the RNA at both 5′ and 3′ ends. Capping represented by solid yellow and leader sequence indicated by L are at 5′-end. ORF, Open reading frame; UTR, Untranslated region (created with BioRender.com. Accessed on 28 July 2021).

As in Figure 3, the genome of the coronaviruses is usually ordered in the following sequence in the 5′–3′ direction: replicase (rep) gene, spike (S), envelope (E), membrane (M), nucleocapsid (N), and accessory genes (interspersed between S and N proteins). The genome is flanked at both ends by untranslated regions (UTRs) [12,15].

A classic coronavirus genome consists of five open reading frames (ORFs): ORFs encoding a 5’-polyproteins (pp): ORF1a/ab, and four 3’-structural proteins: S, E, M, and N [16]. The first ORFa/b (replicase (Rep) gene) encodes 15 or 16 non-structural proteins (nsps) (1–15 or 1–16 nsps). The nsps are required for replication of the virus [12,17,18]. ORF1a/b from the 5′-capped RNA genomes is translated to produce a shorter polyprotein (pp1a, including nsps 1–11) or a longer polyprotein (pp1ab, including nsp1–10 and nsp12–16) based on whether the stop codon is recognized or bypassed at the end of ORF1a, respectively. The minus one (−1) ribosomal frameshifting just upstream of the stop codon in the overlapping region between ORF1a and ORF1b ends up in bypassing the ORF1a stop codon. This directs the process into the production of the larger pp1ab polyprotein [2,17,19]. The presence of a hairpin-like RNA pseudoknot (PK) structure and a heptanucleotide slippery sequence (UUUAAAC) that precedes the PK structure, is responsible for triggering the frameshifting event [19,20]. Both the replication of genome and the production of the nested set of sg mRNAs are mediated by the nsps, which are encoded in the replicase gene and assembled into the membrane associated viral replication-transcription complex (RTC) together with possibly a few numbers of cellular proteins and the viral N protein. From these proteins, RNA dependent RNA polymerase (RdRp) (nsp12); nsp7-nsp8 complex, which is a processivity factor for the RdRp; and helicase (nsp13) are formed. These are the key enzymes involved in coronavirus RNA synthesis [1,19,21].

Coronaviruses are known to encode a distinct set of RNA-altering activities that are not present in other viral RNA genomes. One of them is nsp14 (Exonuclease N, ExoN) proofreading activity, which is linked to the DEDD superfamily of exonucleases. It confers increased fidelity so that it is believed to assist in the replication-transcription process of larger viral genomes [1,19,21,22]. In addition, nsp14 also contains the N7-methyltransferase (N7-MTase) domain in its C-terminal, which is supposed to help in the viral mRNA capping [21,23].

Accessory and structural proteins are coded by other ORFs from the genome’s 3′- end [1]. The accessory proteins vary in number and in a species-specific manner. Among structural proteins, the S protein binding to the relevant receptor of target cell mediates viral entry and then the process of fusion with the host cell membrane is initiated [1,19,24]. The E protein facilitates the viral assembly by forming an ion channel in the membrane [19,25]. The morphogenesis of virus is mediated by the M protein through incorporation of essential viral components into new virions. Association of N protein with M protein and viral genome directs genome packaging into new viral particles [19]. Some coronaviruses, especially some Betacoronaviruses may also have a second peripheral short 5–10 nm long hemagglutinin esterase protein spike [13,26]. Interspersed between structural proteins are accessory proteins, which are suspected to have important roles including the interferon (IFN) signaling pathways and pro-inflammatory cytokine production; although they are considered dispensable for virus replication in cell culture [1,27].

While replication of coronavirus is continuous, transcription is discontinuous [21]. The positive-sense genomic RNA copies are synthesized during replication [1,2,12,19]. These mRNAs are translated to more nsps and replication transcription complexes are assembled into new virions [1,19,28]. In the process, diverting host endomembrane into replication organelles has been hypothesized to be mediated by membrane-spanning nsp3, nsp4, and nsp6. Subsequent discontinuous transcription and translation into various proteins helps run the life cycle of coronaviruses [1,12,19,29,30].

The family Coronaviridae is further classified into the subfamily Orthocoronavirinae consisting of four genera or groups (1–4): Alphacoronavirus, Betacoronavirus, Gammacoronavirus, and Deltacoronavirus. While Gammacoronavirus and Deltacoronavirus include avian and animal coronaviruses, causing respiratory and some other types of diseases; Alphacoronavirus and Betacoronavirus consists of human and animal coronavirus infections that mainly result in respiratory and enteric diseases, posing a challenge for public health by causing severe outbreaks and even pandemics. As the latter have affected livestock and companion animals, they are veterinary and economic concerns as well [1,2,11,12,19,31,32].

## 3. Human (Pathogenic) Coronaviruses

Two Alphacoronavirus strains (HCoV-229E and HCoV-NL63) and five Betacoronaviruses (HCoV-HKU1, HCoV-OC43, SARS-CoV, MERS-CoV, and SARS-CoV-2) have so far been recognized as HCoVs [2,12,19]. HCoV-229E, HCoV-NL63, HCoV-OC43, and HCoV-HKU1 which are circulating in endemic forms worldwide in the human population [2,12,33], commonly cause 10–30% cases of common colds globally and are known to cause mild upper respiratory illnesses [19,34]. Two zoonotic HCoVs: MERS-CoV and SARS-CoV along with recently emerged SARS-CoV-2 are highly pathogenic strains [2,12]. These strains have been thought to have emerged into the human population from wildlife through spillover events, causing severe illnesses of the respiratory tract especially the infections of lower respiratory tract. SARS-CoV appeared in 2002, MERS-CoV in 2011, and SARS-CoV-2 was first reported in 2019, with its devastating attack against humanity still in existence [19,35]. Recent sequence database survey analysis revealed that all HCoVs originated from animals: HCoV-NL63, HCoV-229E, SARS-CoV, and MERS-CoV from bats, and, HCoV-OC43 and HCoV- HKU1 from rodents [12,36,37,38]. In addition to the natural/reservoir host, intermediate hosts are required for the transmission of the many viruses from reservoir hosts to humans [12,18,39]. Palm civets, bovines, and dromedary camels are the intermediate hosts confirmed for SARS-CoV, HCoV-OC43, and MERS-CoV respectively [18,33,40]. Similarly, HCoV-229E is more closely related to viruses circulating in camels than to those in bats, suggesting camelids as the possible intermediate host between bat and human [41]. Interestingly, as amino acids sequences at the receptor binding domain (RBD) of the S protein of SARS-CoV-2 and coronavirus strain isolated from pangolins displayed 97.4% homology and 5 identical amino acid residues, the latter are most likely the intermediate host of the SARS-CoV-2 [18,33,42]. Intermediate hosts for the remaining two HCoVs are unknown so far [19,40]. The comparative properties of seven HCoVs and associated diseases have been summarized in Table 1 below.

## 4. Transmissions

Human-to-human transmissions of coronaviruses can occur via droplets, contaminated hands, or surfaces. These viruses can remain infectious on inanimate surfaces for up to nine days [48]. SARS-CoV-2 can go directly from person-to-person, enabling their spread by respiratory droplets, face-to-face contact, and fomite [4,49]. Surprisingly, pre-symptomatic carriers cause an estimated 48–62% of SARS-CoV-2 transmission [50]. Inhalation of virus, deposition of virus on exposed mucous membranes, and touching mucous membranes with soiled hands contaminated with virus are the key routes of SARS-CoV-2 transmission. Notably the risk of SARS-CoV-2 infection has been reported to differ according to the volume of virus to which a person is exposed. Recently it has also been noticed that transmission of SARS-CoV-2 from inhalation of the air droplet farther than six feet from a person to person can also be possible [51].

## 5. Host-Virus Interactions

After adherence of viral S protein to the host cell receptor marking the initiation point of coronavirus infection, the host protease cleaves the S protein into S1 and S2 subunits which represents the N-terminal receptor binding domain and C-terminal domain of S protein, respectively [2,12,52]. Transmembrane surface serine protease 2 (TMPRSS2) helps in this process in case of SARS-CoV-2 [1,2,53]. However, in SARS-CoV, endocytosis mediated entry may possibly involve the endosomal cysteine protease Cathepsin B and L (Cts B and Cts L) [1,2,54]. During the binding and entry process, the virus-cell tropism and pathogenicity are determined by the RBD found in the surface exposed S1 subunit, which engages the host cell receptor. Similarly, heptad repeat (HR) regions: HR1 and HR2, and the fusion peptide (FP) contained in the transmembrane S2 domain mediate extensive conformational rearrangements [1,2,12,55,56]. For this, the formation of a six-helix bundle (6-HB) fusion core mediated by the interaction between HR1 and HR2 brings two cell surfaces in proximity. This rearrangement or change facilitates fusion of virus and the host cellular membrane, which permits the passage of essential nucleocapsid into the host cellular cytoplasm by facilitating correct lipid bilayer orientation and location [2,12,13,18,57,58,59]. Even after the separation of S2 from S1 by the first cleavage event, the two domains keep their association non-covalently. The second event of cleavage at S2 is compulsory, which leads to the final fusion of the viral and cellular membranes enabling the actual and complete release of the N protein-coated viral RNA genome into the cytoplasm [2,18,19]. During the first cleavage of S protein in the case of SARS-CoV-2, the efficient cleavage by the prototype protein convertase (furin) is believed to be mediated by the acquisition of a polybasic cleavage site (PRRAR) at the S1–S2 boundary [1,12]. In the process, proline insertion at the junction of S1 and S2 subunits may help in incorporation of O-linked glycans to the residues within cleavage site by the formation of a stem-loop structure. As both the effective infection and overcoming the species barriers might have been influenced by efficient S protein cleavage; such a cleavage can be proposed to be the reason for enhanced infection and even the evolution of SARS-CoV-2 [1,12,19,60,61,62,63]. Additionally, relatively increased cell tropism, enhanced zoonotic potential, and heightened transmissibility of SARS-CoV-2 other than HCoVs may be caused by this furin mediated pre-processing of the SARS-CoV-2 S protein [1,2,12,19,64,65]. After endocytosis, lipid bilayers are taken apart by lysosomal enzymes which are produced by type II pneumocytes. Next, the virus uses the host cell RNA polymerase and ribosomes for transcription of viral RNA, and the viral load within the host cell is increased [3].

**Figure 4:**
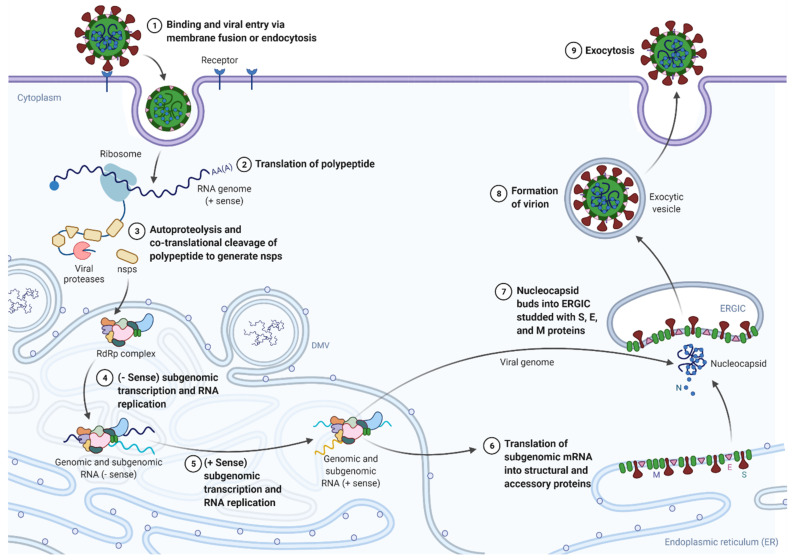
Major events in the life cycle of coronaviruses. Nsps, non-structural proteins; DMV, double membrane vesicles; ERGIC, endoplasmic reticulum-golgi intermediate compartment (created with BioRender.com. Accessed on 28 July 2021).

For mRNA translation into Rep pp, the genomic RNA acts as mRNA [12]. In this process, synthesis of PP1a and PP1ab is performed by the expression of the ORF1a and ORF1ab, respectively, within the Rep gene [1,12,66]. Papain-like protease (PLPro) which is contained or encoded by gene in nsp 3) brings about autoproteolytic cleavage of pp1a (polyprotein 1a) so that 11 nsps (non-structural proteins) are formed. Similarly, Chymotrypsin-like protease (3CLpro) (which is encoded by the genes in nsp 5) brings about autoproteolytic cleavage of pp1ab (polyprotein 1ab) so that 15 nsps are formed [1,12,18,19,67]. A subset of nsps is thus generated and comes together to create the RTCs that reside in convoluted membrane structures derived from the rough endoplasmic reticulum (ER) of the host cell. Viral transmembrane proteins provide the anchorage for the resulting structure. The assembled RTCs can copy and transcribe the genomic RNA [1,19,68,69]. Rep utilizes genomic RNA in place of a template for synthesizing negative sense RNA (−RNA) and −RNA acts as a template for positive sense RNA (+RNA) [1,2,12,21]. Consequently, discontinuous transcription enables Rep to produce a nested set of sg mRNAs [1,2,12,21,70]. The detailed process of coronaviral life cycle has been depicted below in Figure 4.

Upstream to the majority of ORFs located in the one-third of the viral genome at its 3′ end are the specific sites called transcription regulatory sequence (TRS). These are the sites where the RdRp complex terminates or pauses during −RNA synthesis. The interaction between the nascent −RNA (TRS body or TRS-B) and the +RNA (TRS-Leader or TRS-L) is the initiation point of discontinuous coronavirus RNA synthesis, which is believed to be mediated by RNA−RNA, RNA-protein, and protein-protein interaction maintained at a certain range of physical proximity. After the TRS-B has been copied, if a minimum threshold of the free energy (ΔG) of duplex formation between the complementary TRS-B and the TRS-L is exceeded, switching of templates takes place. The template switching is jumping off TRS-B to the TRS-L of the viral genome, inserting a copy of the TRS-L. Copying of the L sequence completes the synthesis of −sg RNA [1,12,19,21,28,30,71,72], which subsequently serves as templates to generate +sg mRNAs by transcription process [1,2,19,21,70]. A number of accessory proteins and transmembrane structural proteins such as M, S, and E are then translated from +sg mRNAs and then inserted into the ER for folding.

During the aforementioned processes of replication and transcription, formation of intermediate double-stranded RNA as well as double membrane vesicles (DMVs), convoluted membrane (CMs) and small open double membrane spherules (DMSs) are hypothesized to be important to maintain the conductive and protective microenvironment inside host cell. The proteins are then carried to ER-Golgi intermediate compartment (ERGIC) [1,2,12,19,21,59]. By that time, the N protein is synthesized by translation of N gene so that progeny genomic RNA can be encapsulated with the N protein. Simultaneously, protein-protein interactions are enabled by M proteins to occur inside ERGIC, which paves the way for the assembly of complete virion [1,2,12,13,19,21,59]. Secretory pathways of the smooth wall vesicles or golgi sacs are activated for the transport of the virions which are newly formed. Then, the fusion of vesicles with the plasma membrane takes place, which sheds out the mature virus particles through budding or exocytic pathways from the infected cell. S proteins of the released viruses, which enable them to infect the nearby host cells, resulting in the increase in the number of infected cells. Viral production inside the cell would then be amplified [2,12,13,19,21,59,73].

## 6. Immune-Evasion Strategies

Glycosylation of viral structural proteins, formation of DMVs, and reduction of ER-lipid content leads to ER stress during coronaviral infections [12,59]. ER stress can also be the result of the upregulation of glucose-regulated protein 94 and 78 (GRP94 and GRP78) [12,74,75]. Stress response factors such as inositol-requiring kinase 1 (IRE1), protein RNA-like ER kinase (PERK), and activating transcription factor 6 (ATF6) are brought into action by unfolded protein response pathway, which is induced by ER stress. This event attempts to maintain the cell homeostasis either by translation attenuation, or by apoptosis. Sometimes during the process of homeostasis, kinase activities believed to be mediated by IRE1 may also help infected host cells to escape the apoptosis induced by viral infection [12,59,76,77,78]. In addition, protecting epitopes from neutralizing antibodies by heavily glycosylated S proteins of coronaviruses may be another mechanism to facilitate immune evasion [1,79,80].

The host response to virus and clearance of viral infections from host greatly rely on Type I IFN (T1IFN) expression [81]. SARS-CoV nsp1, which is considered as one of the host’s shut-off switches or pathogenicity factors, binds to the smaller 40S subunit of ribosome so that it induces degradation of host mRNAs including that of IFN. In this way, nsp1 can suppress the host’s protein translation [19,82]. In addition, nsp3 and structural proteins (M and N) of MERS-CoV and or SARS-CoV may work as the antagonists for IFN pathways [83,84]. Retinoic acid-inducible gene I (RIG-I)-like receptors and Toll-like receptors (TLRs) collectively known as pattern recognition receptors (PRRs) are also found to be inhibited by coronaviruses, eventually leading to un-productive inflammation as the related signaling is indispensable in many immune pathways [85,86].

As long as SARS-CoV-2 can employ unique 2′-O-methyltransferase capping machinery to shield its RNA from cellular innate immune recognition, this can also be a convincing way of immune evasion by SARS-CoV-2 [1,21,87]. The enzyme 2′-O-methyltransferase that inserts 2′ O-methyl group to the RNA of virus is also produced by SARS-CoV, which helps it escape detection by melanoma differentiation associated protein 5 (MDA 5). Induction of IFN is delayed by this enzyme, which may cause an inability to control viral replication, resulting in the enhanced infection, cellular damage of airway epithelia and the lung parenchyma, along with an eventual lethal inflammatory cytokine storm [2,88,89]. Another enzyme: PL^Pro^ produced by SARS-CoV-2 is required to generate the RTC. For this purpose, IFN stimulated gene 15 (ISG15), which is a ubiquitin-like protein, is cleaved from interferon responsive factor 3 (IRF3) by the help of PL^Pro^ enzyme. This cleavage attenuates the T1IFN responses [1,90]. Activation of mitochondrial antiviral-signaling (MAVS) proteins is inhibited by SARS-CoV. MAVS protein is considered indispensable for the nuclear translocation and activation of IRF3 in reaction to the activation of cytoplasmic RNA sensor. As tissue necrosis factor receptor-associated factors 3 and 6 (TRAF3 and TRAF6) are key actors for the induction of IRF-3/7 in response to RIG-I and MDA 5 ligation, TLR3/7 and nuclear factor kappa B (NF-κB) signaling pathways, inhibition of TRAF 3/6 by SARS-CoV and also possibly by SARS-CoV-2 are consequential in evading the host immune system [33,91]. The phosphorylation of signal transducer and activator of transcription (STAT) pathway may be inhibited by SARS-CoV-2, which can be a mechanism to offset T1IFN signaling [33,92]. This enables the virus to propagate without stimulating the innate anti-viral response machinery of the host. Even if infected cells undergo cell death, releasing virus particles in the later stage, virus particles are recognized by PRRs and this event induces the expression of inflammatory cytokines and adaptive immune cells, so that induction of T-cell apoptosis may still be the feasible strategy of SARS-CoV-2, enabling it to partially evade the immune response [33,93]. Similarly, reduced effector T-cell function and decreased proliferative capacity may be caused by CD8+ T cell exhaustion, which by itself is due to chronic viral infections, altogether with cytokine production. CD279 (PD-1) is one of the mature T cell checkpoints for the initiation of apoptosis. T cell exhaustion induces the overexpression of such types of inhibitory receptors. After the binding of PD-1 to its ligands (PD-L1) and PD-L2, the functions of T-cells, B-Cells and NK cells are altered and as a consequence, the immune system can be suppressed [4,94,95].

## 7. Pathogenesis

Type II alveolar cells (or pneumocytes) producing surfactant, and goblet and ciliated cells in the airways are expected to offer a viral portal of entry in humans as the host receptors for, e.g., ACE2 for SARS-CoV-2, are relatively highly expressed on these cells [33,96,97]. Based on WES data on ACE2 variants of Italian COVID-19 patients, allelic heterogeneity in conformation of ACE2 may be the basis of difference in individuals in-terms of susceptibility to virus entry, disease onset, and progression [98]. ACE2 receptors are also expressed by nasal ciliated and goblet cells so after the inhalation of viral particles present in respiratory droplets from an infective host, viruses bind the receptors and go to the interior of the nose of a healthy host [33,97]. The virus encounters only a limited innate immune response at this time. The virus keeps maintaining the slow pace of replication until it goes down towards the conducting airways of the respiratory tract. A relatively robust innate immune response can be induced as the cellular PRRs like TLRs, RIG-1, and MDA 5 detect the virions while the proliferating virions begin circulating through the upper respiratory tract. Expression of T1IFN in the early stages of infection is resulted immediately after the recognition of virions by PRRs. Now, the generation of inflammatory cytokines and chemokines marks the establishment of an anti-viral state in the infected cells [33,88,89,99]. Excessive edema and granulations in the upper trachea and subglottis, ulceration of the subglottis and epiglottis, and dysphonia (hoarseness) are often observed as the main symptoms in the patient. Mild tachypnoea and coarse breath sounds can also be found in a few patients while the virus is in the upper respiratory tract [33,100,101].

After the virus reaches the lower respiratory tract and proceeds all the way down to the lungs, various inflammatory mediators are likely to be released after the SARS-CoV-2 buds off and subsequently damages host cells such as the type II pneumocytes. Activated alveolar macrophage produces tissue necrosis factor-alpha (TNF-α), interleukin-1 (IL-1) and IL-6. These cytokines enter the vascular system triggering further responses. Collectively, they increase capillary permeability by two ways: by the contraction of blood vessel endothelial cells and dilation of smooth muscle. This leads to the leakage of plasma from the blood vessel into the interstitial spaces. Alveolar edema is resulted by those series of events [3,33,102,103]. IL-8 is also released by infected lung epithelial cells. The IL-8 functions as a chemoattractant, recruiting T lymphocytes and neutrophils to the site of infection. As neutrophils are swiftly recruited to the sites of infection, secretion of defensins, an oxidative burst, and the neutrophil extracellular traps (NETs) are the mechanisms employed by neutrophils to destroy the viruses [4,104]. Both types I and II pneumocytes, whether infected or non-infected, are destroyed by reactive oxygen species (ROS) and proteases released by activated neutrophils. Destruction of types I and II pneumocytes lead to reduced gas exchange and alveolar collapse, respectively, along with alveolar/pulmonary edema. The second clinical presentation is correlated with increased surface tension, which by itself, is associated with decreased production of surfactant [2,104]. Additionally, fluid, protein deposits, cell debris, macrophages, and neutrophils from the dead pneumocytes are collected into alveoli collectively, resulting in the pulmonary consolidation, which is characterized by the altered gas exchange and ultimately leads to the hypoxemia [2,105]. Similarly, IL-17, secreted mainly by T-helper cell 17 (Th17), stimulates production of IL-1, IL-6, IL-8, monocyte chemoattractant protein 1 (MCP-1), growth-regulated oncogene alpha (GRO- α), granulocyte-colony stimulating factor (G-CSF), granulocyte macrophage colony stimulating factor (GM-CSF), TNF-α, and prostaglandin 2, which can induce the recruitment of neutrophils, monocytes, and other immune cells. IL-17 expression has also been reported to be correlated with several inflammatory respiratory diseases, besides being associated with autoimmune conditions [106,107]. In the mucosal immune response, IL-17, IL-22, and TNF-α produce antimicrobial peptides. In addition, IL-22, which exhibits mucosal immune response along with IL-17 and TNF-α, upregulates mucins, fibrinogen, and anti-apoptotic proteins so that it may contribute to the development of pulmonary edema, which itself may be severe sequelae. The progression of acute respiratory distress syndrome (ARDS) may be caused by enrichment of alveolar tissue with mucins and fibrin in the lungs [4,107]. Further buildup of pathogenic inflammatory cells may also be possible through the stimulation of IFN receptors, generation of chemokines such as CCL2, CCL7, and CCL12, and build-up of inflammatory cytokines such as TNF, IL-6, and IL-1β [3,108,109].

Macrophage activation syndrome (MAS) is associated with cytokine storm (CS) [110], which is characterized by abnormal levels of chemokines and cytokines, both pro and anti-inflammatory ones, such as IL-1, IL-2, IL-4, IL-6, IL-7, IL-10, IL-12, IL-13, IL-17, IFN-γ, TNF-ɑ, G-CSF, GM-CSF, macrophage inflammatory protein-1 alpha (MIP-1α), IFN-γ  induced protein-10 (IP-10), monocyte chemoattractant protein-1 (MCP-1), hepatocyte growth factor (HGF), and vascular endothelial growth factor (VEGF) [111]. CS may feature both ARDS and pneumonia [112]. Besides multi-organ failure in the heart, liver and kidneys, hyper-ferritinemia, and coagulopathy, usually presented with higher level of blood urea nitrogen (BUN), D-dimer, C-reactive protein (CRP), and Creatine (Cr) are characteristic of not only MAS, but also of secondary Hemophagocytic lymphohistiocytosis (sHLH) [4]. Similarly, hyperinflammation triggers lung injury along with the severe form of alveolar edema and bilateral diffuse alveolar tissue damage. Mechanistically, fibrinolysis as well as increased thrombus generation accumulates mucins and fibrin in inflammatory sites, which is responsible for pulmonary edema. The sequence of these events unfortunately causes severe respiratory failure and death in severely affected patients [4,113,114,115,116]. Bilateral lung involvement with ground-glass opacities seen in computed tomography (CT) scanning of the chest is the most common diagnostic feature in the severe cases of COVID-19. An inflammatory syndrome seen with the disease progression can also resemble septic shock. Similarly, the formation of hyaline membranes can be revealed by histological examination of the lungs [116,117].

Viral single stranded RNA is a pathogen associated molecular patterns (PAMPs) of SARS-CoV-2, which is recognized by various cellular PRRs. Dendritic cells and monocyte-macrophages express TLR7 as a PRR so that TLR7 can initiate a strong innate immune response in terms of several signaling pathways and transcription factors, such as Janus kinase (JAK), STAT, NF-κB, activator protein 1 (AP-1), IRF3, and IRF7 [4,118]. In a recent study, COVID-19 susceptibility in severely affected young male has been associated with missense deleterious variants in the X-linked recessive TLR7 gene. Further study on this loss-of-function mutation can elucidate another mechanism for relatively higher susceptibility of males to COVID-19 disease [9]. Similarly, less favorable outcome in COVID-19 has been associated with poly-glutamic (PolyQ) repeat number of Androgen receptor (AR) and serum testosterone concentration. This fact, though cautiously, suggests the potential of the shorter polyQ (≤22) and testosterone hormone as adjuvant for COVID-19 treatment [119]. Normally after infection, the lung epithelial cells, and neutrophils preferentially initiate the innate immune response. In the next stage, specific adaptive immune responses (both humoral and cellular immunity) are initiated [4,120], which culminates in approximately 7–14 days after infection. Following the presentation of antigens by antigen presenting cells (APCs) to the CD4+ and CD8+ T-cells, pro-inflammatory cytokines are generated via the NF-κB signaling pathways. Then, virus-specific antibodies, likely to mount a neutralizing effect, are produced by activated B cells. In addition, antigen-specific T cytotoxic cells may be produced to destroy virus-infected cells. These complex events are the body’s attempts to control SARS-CoV-2 in the human tissue [4,114,121].

During viral infections, more healthy cells are targeted by CS, which is manifested by the elevated production of chemokines and inflammatory cytokines from monocytes and neutrophils in the lung tissues and peripheral blood of patients [3,122]. A higher expression level of genes encoding proinflammatory cytokines such as IL-2, IL-7, IL-10, G-CSF, MIP-1α, and TNF-α may bring about CS [123]. While an increased expression of IL-6 and IL-8 are already known to be involved in ARDS, many candidate genes, i.e., ACE2, IL-10, TNF, and VEGF are also being studied as they are believed to be associated with ARDS development or outcome [124].

Reduced production of the surfactant caused by the destruction of types I and II pneumocytes is associated with the surface tension within the alveolus. This contributes to alveolar edema in addition to alveolar collapse [3,103]. The gas exchange is compromised with resulting alveolar collapse. Then, refractory hypoxemia is developed, and peripheral chemoreceptors are stimulated. The sympathetic nervous system stimulated by the chemoreceptors attempt to compensate the reduced gas exchange by increasing the respiration and heart rate. The patient’s breathing is impaired with severely reduced partial pressure of oxygen, ultimately leading to ARDS [3,102]. Production of neutralizing antibodies are also thought to be responsible for the organ damage and poor outcome during the infection. In this context, through their binding to Fcγ receptors, antibody-dependent enhancement (ADE) has been found to boost the uptake of virus particles bound in immune complexes of the host cell. Persistent viral replication intensified with ADE in immune cells such as APC that is newly infected, and the inflammatory responses mediated by immune-complex, eventually causes tissue and organ damage, along with ARDS [33,125,126]. The hypothalamus of the brain is stimulated with increased levels of vascular cytokines: IL-1 and IL-6. Simultaneously, higher prostaglandins E2 production contributes to raise the core body temperature from normal temperature, initiating the fever. Abnormally elevated levels of cytokines circulating through the vascular system may reach other tissues sparking the systemic inflammatory response syndrome [3,102]. In terms of systemic infection, atrophy of the lymphatic tissues such as spleen and lymph nodes, and lymphopenia are common in the case of severely ill COVID-19 patients experiencing CS [127]. Similarly, death of inflammatory cell and lymphatic organs’ hypo-cellularity are common features of primary, and secondary forms of HLH (pHLH and sHLH) and associated CS [3,33,128]. Histopathologic reports from tissue sections from a subset of COVID-19 patients suggest that infarction, wall thickening, and focal hemorrhage are potential features of vasculitis. Vasculitis lesion is mediated by deposition of immune-complex and it is accompanied by occlusion or infiltration of lymphocytes and monocytes around and within the blood vessels [33,129,130].

Similarly, blood volume can be reduced as the capillary hyperpermeability induced by vascular cytokines can direct plasma to move towards deposits within tissues other than the lungs. As the total peripheral resistance is reduced by vasodilation, and blood pressure is lowered, exhausted perfusion can be the next significant sequelae in the body, which ultimately results in the multi-system organ failure (MSOF) [3,122]. Elevated levels of BUN and Cr are found to be correlated with the diminished perfusion to kidneys, ultimately leading to acute renal injury. Besides, disseminated intravascular coagulation (DIC) has also been found to be associated with a severe feature or complication in some COVID-19 cases [3,131]. Data from the earlier studies in Germany showed that lymphopenia and thrombocytopenia along with the increase in CRP, lactate dehydrogenase (LDH), and D-dimers were associated with a less favorable outcome for the patient. Although the troponin level was also found to be increased, its significance was unclear [132].

## 8. Potential Mechanisms of Co-Morbidities in COVID-19 Disease

As the pancreatic islet cells overexpress ACE2, diabetogenic SARS-CoV-2 may instigate severe imbalance in the blood glucose levels of sugar patients. This may worsen the inflammatory instability [133,134] suggesting the diabetic morbidity in COVID-19 infection. Higher expression of ACE2 in a normal adult has been described as the reason for the higher rate of infections in adults as compared to children [4]. Contrastingly, it has also been reported that expression of ACE2 is the highest in young women and children; the lowest in people suffering from chronic disease, such as sugar, obesity, and high blood pressure; and it declines with age, which links the ACE2 expression inversely with risk of unfavorable outcomes after severe presentation of disease [33,134]. In the latter view, beside the role of ACE2 in facilitating the binding and entry of SARS-CoV-2, it also plays a role in regulating both inflammation and infections. RAS is required to maintain fluid and electrolyte balance in blood, and ACE2/angiotensin/Mas balance is the critical component of RAS (Figure 1). In RAS, ACE2 catalyzes angiotensin-II processing into angiotensin [33,135,136,137], which counteracts vasoconstriction (favors vasodilation) by directing the pathway into MasR (anti-fibrotic) arm of RAS rather than to the pro-inflammatory/pro-fibrotic angiotensin type 1 receptor (AT1R) one. Thus, limiting tissue inflammation while favoring repair mechanisms is the proposed role of ACE2 but warrants further experiments to either support or deny the hypothesis [33,137,138].

## 9. Animal/Virus Models for Research on HCoV Infections

Finding the perfect animal model that can replicate underlying etiopathogenesis and clinical features of COVID-19 is a pressing need of our time to scientifically fight the pandemic. The assessment of underlying mechanisms, potential vaccines, and therapeutic strategies are part of the tasks required to address the exigency created upon the scientific world. Such endeavors are not possible without such ideal animal models [116,139,140,141]. Different sites and organs of mice, ferrets, hamsters, and non-human primates such as cynomolgus and rhesus macaques facilitate the replication of SARS-CoV-2. These animals are also found to display at least one of the clinical symptoms of SARS-CoV-2 infections. Larger animal models, such as ferrets, to some extents are ideal for reproducing the COVID-19 pathology of humans [141]. In a study, viral antigens were detected in nasal turbinate, trachea, lungs, and intestine along with acute bronchiolitis present in infected lungs as well as the elevated temperature of the body of ferret. Airborne transmission of SARS-CoV-2 from infected to naïve ferrets were also reported [142]. However, as the small animals, such as mice and Syrian hamster, reproduce faster, they can also be a better model for studying SARS-CoV-2. Rapid breathing, weight loss, and alveolar damage with extensive apoptosis, which are almost the same human symptoms upon SARS-CoV-2 infection, were also displayed by hamsters [140,141]. Though SARS-CoV-2 cannot use mouse ACE2, transgenic (genetically modified) mice that express human ACE2 were found to replicate human SARS-CoV-2 pathologies. In a study, a transgenic mouse showed replication of virus in the lungs, interstitial pneumonia and loss of weight, mimicking the infection in humans [140,141,143,144]. Alternatively, as the populations of RNA viruses consist of a swarm of closely related viral quasi-species, even the rare viruses in the swarm that contain mutations in the spike protein that increase their binding affinity to mouse ACE2 are expected to be selected. Given their higher levels of replication in mouse lungs tissue, sequential passaging of SARS-CoV-2 can be a good strategy to adapt SARS-CoV-2 to mouse ACE2 so that further study can be feasible with the adapted virus using normal mice [116,139]. Although, SARS-CoV-2 infection of humans can be effectively replicated in animals, understanding the possible different results between human and non-human (animal) models might be complicated by the genetic diversity. In this context, humanized animal models such as the mice engrafted with one or more human tissues or cells (for, e.g., mice having human lung engraftment), are part of the renewed options for directly studying the coronavirus infection in human organs or tissues. These can also be an effective tool for the evaluation of vaccines or drugs targeted to specific organs against coronavirus infections [116,140,141]. The pathology of COVID-19 can be reproduced and observed in a tissue-specific and systemic manner in animal models. In addition, cell lines and organoids can be a faster and alternative preclinical model system to study the virus and its interactions inside host cells, as they are not only ethically more feasible, but also already proven to be better at reproducing the COVID-19 symptoms in the context of specific cell types or organs, respectively [141]. On the other side of possibility, the extensive molecular sequence alignment uncovered 54% homology among various coronavirus strains, with higher conservation or identity shown by nsps [12,145]. Improved prevention and control strategies for zoonotic coronaviruses likely to emerge in the future can be gained by better knowledge of the coronaviruses infecting companion animals, their ability for transmission in cross-species manner, and the sharing of relevant molecular biological information [146]. Notable coronaviruses that can infect companion animals include, but are not limited to, feline enteric coronavirus, equine enteric coronavirus, feline infectious peritonitis virus, ferret enteric coronavirus, ferret systemic coronavirus, alpaca respiratory coronavirus, canine enteric coronavirus, canine respiratory coronavirus, and alpaca enteric coronavirus. Among them, feline infectious peritonitis virus, feline enteric coronavirus, canine enteric coronavirus, ferret enteric coronavirus, ferret systemic coronavirus, and alpaca respiratory coronavirus are Alphacoronaviruses, while the remaining canine respiratory coronavirus, equine enteric coronavirus, and alpaca enteric coronavirus are Betacoronaviruses [146,147].

## 10. Antiviral Drugs against SARS-CoV-2

COVID-19 vaccines are now being employed as an indispensable strategy to curb the pandemic, but the people who are unvaccinated, whose bodies do not mount a strong response to the vaccine and who experience breakthrough infections may need antiviral drugs [148]. Optimizing different treatments for mild, moderate, severe and critical illnesses caused by COVID-19 is a unique caveat in the fight against pandemic [149]. However, as there are no universally approved antivirals, it implies the fact that all the antivirals listed in Section 10 are newly proposed drugs. If ongoing trials or studies proves any of them effective in treatments, they can get full approval everywhere throughout the world.

Antiviral agents can be effective only if they are used well before the severe inflammatory response is mounted, and depends on the targeted mechanism [150]. In the following sections, antivirals including repurposed ones, which have already demonstrated some sort of promising efficacy against SARS-CoV-2 either in vitro or in animal studies, will be discussed.

### 10.1. Convalescent Plasma (CP) Therapy

CP therapy involves the blocking of virus infection to ensure the clearance of virus, for which sera containing anti-SARS-CoV-2 antibodies are given to patients. Patients who have been already infected and recovered from COVID-19 and also currently harbor high neutralizing antibody titers against SARS-CoV-2 are the donor source of CP therapy [18,151]. As the CP therapy was found effective in the treatments of patients infected with SARS-CoV in 2002–2003 and with MERS-CoV in 2011, CP is now considered as an alternative for COVID-19 treatment, but it may also have practical implications [18,150].

### 10.2. Remdesivir

Remdesivir is an adenosine nucleotide prodrug that is capable of inhibiting RNA polymerase of coronaviruses even in the presence of the proofreading activity of 3′–5′ exoribonuclease, which enzymatic action is supposed to be problematic for many other drugs. It is inserted covalently into the first replicated base pair through the primer strand. As the central network of the RdRp is occupied with the partial double-stranded RNA template, elongation of the chain during viral RNA replication is terminated [150,152]. The compassionate-use study for this drug was concluded with the result that remdesivir might offer clinical benefit in the severe cases of COVID-19 [150,153].

### 10.3. Favipiravir

Favipiravir is a broad-spectrum anti-viral drug that is potentially promising for specifically untreatable RNA viral infections. Chemically, it is a purine nucleotide analogue. The drug acts as a substrate and inhibits RdRp for which an active favipiravir-ribofuranosyl-5′ -triphosphate (RTP) form of Favipiravir is made through its intracellular phosphoribosylation [18,150,154]. Despite the encouraging profile of favipiravir in terms of effectiveness for treating COVID-19 patients, there is a concern with favipiravir being slower to work especially in the severe patients is the concern [150].

### 10.4. Chloroquine/Hydroxychloroquine

Chloroquine and hydroxychloroquine are thought to attach to sialic acids with high affinity in the lungs so that they can spread across cell membranes to lysosomal vesicles in the cytoplasm. As the drug is trapped in the lysosome of cell, the cell increases its pH (decreasing its acidity). As the cell environment becomes more acidic, the cell is now unable to perform certain functions such as endocytosis, exosome release, or phagolysosome fusion. As a result, the cleavage of viral S protein cannot occur, which is a crucial stage of infection [150,155]. Efforts to recommend hydroxychloroquine as a therapy for COVID-19 have so far failed. However, the potential benefits of hydroxychloroquine remain to be determined [150].

### 10.5. Azithromycin

Though Azithromycin is a macrolide antibiotic, increased pH primarily caused by its intracellular accumulation can also impair the function of both lysosome and trans-golgi network. So, it could interfere in the process of SARS-CoV-2 binding to respiratory cells. Though, it seems useful only in the beginning days of COVID-19 infection, either azithromycin alone or in combination with hydroxychloroquine, could possibly minimize both the requirement for hospitalization or time for clinical recovery [156,157,158].

### 10.6. Ivermectin

Ivermectin is principally an antiparasitic drug. However, it is believed to target the host nuclear transport importin alpha/beta1 heterodimer and it has also been shown to block the in vitro replication of many single-stranded RNA viruses [150,159]. Although replication of coronaviruses takes place in the membrane-protected vesicle within the cytoplasm, the host cell nucleus has also been known to contain some proteins of coronaviruses so that they are projected to be the possible target of ivermectin’s activity [150,159,160] suggesting the potential efficacy against SARS-CoV-2 as well.

### 10.7. Lopinavir/Ritonavir

Lopinavir (LPV) is a protease inhibitor against HIV-1 that functions by blocking the release of mature virions into new cells. It is given in combination with ritonavir (r) which through the inhibition of Cytochrome (Cyt) P450/CYP3A-mediated the metabolism of LPV, increasing the LPV’s plasma half-life. This is likely to enhance the LPV’s pharmacokinetic and pharmacodynamic profile in-terms of its anti-viral activity and exposure [18,33,161]. The effectiveness of treatment with a combination of LPV/r, IFN-β1, and ribavirin for 14 days was assessed against only LPV/r (Control) in a prospective, open-label, multi-center, randomized trial with 127 human subjects infected with SARS-CoV-2 in Hong Kong. The study showed that viral shedding, symptoms, and duration of hospital stay all were significantly reduced with the combination treatment as compared to the control [162,163].

### 10.8. Darunavir (DRV)

DRV is a HIV-1 protease inhibitor, which has also been shown to have significant activity in blocking the in vitro replication of SARS-CoV-2. DRV in combination with Cobicistat (c) (DRV/c) has been shown to significantly inhibit the replication of the SARS-CoV-2. To improve the pharmacodynamics and pharmacokinetics of darunavir, cobicistat, such as ritonavir in LPV/r, can inhibit the function of Cyt P450/CYP3A and thus works as a booster [163,164].

### 10.9. Viral Proteinase/Protease Inhibitors

3CL^pro^ and PL^pro^ are required for the breakdown of viral polyprotein into functional units that are finally assembled into new viruses within host cell compartments. Pyridine-containing α-ketoamides 13a and 13b are proposed inhibitors of 3CL^pro^ in SARS-CoV-2 with relevant data of X-ray crystallography that have shown positive pharmacokinetic properties in mice. High-throughput screening and subsequent plaque-reduction assay with simian Vero cells have presented N3, a Michael acceptor inhibitor and ebselen, an organoselenium compound as potential antivirals targeting 3CL^pro^ [165,166]. Similarly, mycophenolic acid is also found to target PL^pro^ [167]. Any inhibitors targeted to proteinase not only block viral replication by preventing cleavage of the nsp3, but also impede nsp3 from not only interfering the IRF3 and NF-kB innate immune pathways of host through deISGylation and deubiquitination but also by eliminating ISG15 from proteins involved in the Jak/Stat pathways. Nsp3 has also been proven to be a component of a molecular pore found in DMV, which is formed in the wake of viral infection and is associated with an embedded structure of nsp3, nsp4, and nsp6 in the rough ER. As the latter structure formed with nsp3 is believed to reshape the DMV, compromised cleavage of nsp3 can block the virus assembly and subsequent release (stage 4 onwards of virus life cycle as depicted in Figure 4) which is occurred by exocytosis and contributes to infection of other cells or other persons [168].

### 10.10. Camostat, Nafamostat Mesylate, and Umifenovir

TMPRSS2 is a cell serine protease found in the alveolar and airway cell that mediates the priming of SARS-CoV-2 S protein and host cell entry driven by S protein (first stage of infection as depicted in the Figure 3). Camostat mesylate is a serine protease inhibitor and has been found to significantly reduce infection with SARS-CoV-2 in human lung Calu-3 cells. Nafamostat mesylate has shown inhibitory activity against the virus in simian Vero E6 cells paving the way for further study [53,169]. Umifenovir also prevents membrane fusion of the viral envelope and host cell cytoplasmic membrane required for virus entry into host cell by inhibition of clathrin-mediated endocytosis [170].

### 10.11. Recombinant Human Soluble (rhs) ACE2

rhsACE2 protein in excess in the soluble form can compete with endogenous transmembrane ACE2 for binding of the S protein of SARS-CoV-2. The exogenous rhsACE2 binds and neutralizes circulating SARS-CoV-2 virions, serving as a scavenger or decoy [171]. During ARDS, the accumulation of the Angiotensin converting enzyme leads to vasoconstriction in pulmonary blood vessels (see Figure 1 for mechanisms), causing a hypoxia-induced lung injury. Conversely, the ACE2 activation has likely a shielding effect against lung damage primarily seen in ARDS, by switching AT1R pathway to the MasR pathway. Therefore, employing rhACE2 could be another encouraging therapeutic approach for severe COVID-19 patients [137,163,172].

### 10.12. Monoclonal Antibodies (mAbs)

Due to their extraordinary specificity to the virus minimizing the risk of off-target effects, exceptional track record of safety in humans, and their ability to organize the immune defense in the fight against viral infections, mAbs are one of the best classes of antiviral molecules under consideration for use against SARS-CoV-2. Specifically, minimizing important pulmonary morbidities associated with the COVID-19 infections by using inhaled/nebulized delivery of antiviral mAb can be an early as well as optimal intervention strategy [173]. However, better early signs of clinical value have been demonstrated by systemic mAb-based therapies, rendering them the more encouraging treatment approaches that likely reduce the progression of SARS-CoV2 into a severe form of pulmonary disease and even hospitalization. This suggests that both type of the mAbs discussed above should be evaluated in parallel. There are two additional challenges prominent so far on the way: optimizing the mAb doses needed to be administered, as well as ensuring such novel treatment to as many patients as possible [173,174]. However, already, the U.S. Federal Drug Administration (FDA) has granted emergency use authorization (EUA) for two mAbs: bamlanivimab (produced by Eli Lilly) and casirimab/imdevimab (produced by Regeneron) that target S protein of SARS-CoV-2 [175].

### 10.13. T1IFN

Given their broad effects, IFNs are often tested for antiviral activities in combination with other agents, such as lopinavir/ritonavir, ribavirin, remdesivir, corticosteroids, or IFN-γ [176]. The in vitro antiviral effect of genetically engineered IFN-α2a, IFN-α2b, IFN-β1a, and IFN-β1b was demonstrated for SARS-CoV, MERS-CoV, and SARS CoV-2. Nevertheless, recombinant interferons were found to affect neither mortality nor viral clearance associated with MERS infection. However, the time of administration could be a factor. Human subjects might not have received treatment early enough during the infection to be fully effective, which can be a factor to be considered for its failure. Further studies are suggested before being certain about their therapeutic fate [33].

## 11. Vaccines against SARS-CoV2

Appreciating the prior experience on humanity to deal with infectious diseases such as mumps, measles, Spanish flu, and SARS; defeating the COVID-19 pandemic by the way of general (mass) vaccination will decrease the economic, social, and psychological pressures already mounted on human society [177]. The vaccines currently available from Pfizer-BioNTech, Moderna, and CureVac are mRNA type, which can be considered the leader of the vaccination program. Similarly, a second type of vaccine (made by Janssen-Johnson & Johnson, AstraZeneca, Sputnik-V, and CanSino) are made using human and primate adenovirus vectors. In addition, the third type of vaccine is available outside of the U.S. These vaccines use an inactivated whole-virus SARS-CoV-2 vaccine (made by Bharat Biotech, Sinopharm, and Sinovac) [178]. Inactivated vaccines (made by killing or weakening the virus) for COVID-19 also work by inducing immune reactions (that rely mostly on antibody) against S proteins (depicted in Figure 2 and Figure 4).

The mRNA vaccines (encased in lipid nanoparticle-LNP) are administered in the upper arm muscle. Once the instructions (mRNA) reach inside the immune cells, the cells use them to make the S protein. After the S protein piece is generated, the cell breaks down the instructions (mRNA) and gets rid of them. Then, the cell displays the protein piece on its surface. As our immune systems recognize that the protein as foreign, building an immune response is begun. Next, antibodies are generated, similarly to what happens in natural infection against COVID-19. Now, our bodies have learned how to protect against future infection. Similarly, in viral vector vaccine, the vector (a different harmless virus) containing gene encoding the S protein piece of the SARS-CoV-2 virus that causes COVID-19, will enter a cell in our body and then use the cell’s machinery to produce a harmless S protein. Then, the cell displays the protein piece on its surface, inducing the immune system [179]. Sinopharm/Sinovac is the representative of a whole inactivated vaccine in which the virus inactivated/killed by beta-propiolactone and absorbed in alum adjuvant, is injected intramuscularly, which produces immunity against future infections [180]. The evaluation of comparative efficacy of the available vaccines can give us the further insight regarding choosing better ones. As live attenuated (weakened virus) vaccines tend to have higher immunogenicity, they imitate natural infection more closely and invoke a wider range of immune responses involving both humoral and cellular immunity as compared to inactivated vaccines [181]. Studies on other vaccine approaches include, but are not limited, to live attenuated as well as protein subunit vaccine platforms, which are also expected to accelerate in the days to come.

## 12. Delta Variant, Other Variants, and Vaccine Effectiveness

The latest, most contagious Delta variant of SARS-CoV-2 has been found in 85 countries so far, targeting mostly the unvaccinated population [182]. COVID-19 cases due to the Delta variant have recently reported to be more than 51% of all cases in the U.S., making the Delta variant the greatest challenge of the pandemic in the country [183] According to CDC director Rochelle Walensky’s senate testimony on 20th July 2021, Delta now accounts for 83% of all sequenced cases in the US, which is a remarkable spike in just two weeks, up from 50%, which was observed in the week of 4 July 2021 [184]. Substitutions/mutations of T19R, (G142D*), 156del, 157del, R158G, L452R, T478K, D614G, P681R, and D950N of gene encoding the SARS-CoV-2 S protein is responsible for molecular evolution of the Delta variant [185]. K417N is associated with the evolution of the Delta plus variant [186]. Mutation is possibly induced by the host environment in terms of the changing immune reaction displayed by humans in response to the infection [187]. The WHO has declared it a Variant of Concern (VOC) and its transmissibility is supposed to be greater by 60% than that of variant Alpha. Sensitivity to certain monoclonal and polyclonal antibodies of members of the B.1.617 lineage (that includes the Delta variant) has also been reported to be reduced as compared to the original strain [188]. A 51–67% higher secondary attack rate than the alpha variant has also been attributed to Delta variant [189].

Either poor or almost no efficiency of a single dose of Pfizer; a mRNA vaccine or AstraZeneca; a viral vector vaccine was shown against Delta variants. However, the second dose of vaccines produced efficient neutralizing response in 95% of subjects targeting variant Delta, although with 3- to 5-fold lower titer than that of Alpha. Similarly, some mAbs, such as Bamlavinimab, were no longer was found to neutralize variant Delta. Sera from naturally immunized individuals was poor in terms of the activity against Delta. However, vaccination of previously infected or convalescent individuals boosted the humoral immune response well above the threshold of neutralization rendering vaccination of those individuals most likely protective against variant Delta and other circulating variants [188].

According to the research of Pfizer, though neither published, nor peer-reviewed, the level of protection provided by the two doses of its COVID-19 vaccine can decrease over time, and a third booster dose may be needed within 6 to 12 months after taking the second dose. The booster dose can enhance antibody levels 5 to 10 times higher over its 2-dose shots. Pfizer is testing both the effectiveness of a third booster dose and is updating its version targeting the Delta variant. However, the Centers of Disease Control and Prevention (CDC) and FDA say that no-vaccination is correlated with virtually all current COVID-19 hospitalizations and deaths in the U.S., therefore, only extensive scientific data can resolve the question of a booster requirement [190]. However, to be on the safe side for potential public health safety, the National Institutes of Health (NIH) has started a Phase ½ clinical trial of administering booster doses of different COVID-19 vaccines after the 12 to 20 weeks of the complete vaccination of adult volunteers to determine the safety and immunogenicity of mixed/matched boosted regimen [191]. In different cases, a third booster of COVID-19 vaccine has been shown to be beneficial in terms of antibody response in a small sample of transplant recipients, which are generally considered immunocompromised patients [192].

Alpha and Beta variants have shown approximately 50% increase in transmission. Similar to these two variants, the Gamma variant, which is the fourth member of VOC designated by WHO, has also shown reduced neutralization by convalescent and post-vaccination sera [193].

## 13. EVs as a New Dimension in Corona Virology

EVs provide a way of intercellular communication by working as carriers of biomolecules (proteins, lipid, mRNA, micro RNA (miRNA), etc.) for transfer among cells [3]. According to the guidelines of the international society of extracellular vesicles (ISEV), three types of vesicles: exosomes (30–120 nm in size; endosomal origin), microvesicles or microparticle or ectosome (100–1000 nm in size; plasma membrane origin), and apoptotic bodies (larger; apoptosis origin) are encompassed in the term EVs. Exosomes from infected host cells have been found to express receptors for SARS-CoV-2, such as CD9 and ACE2, which may be involved in accelerating viral entry and evading immune cell recognition, eventually promoting the infection [194].

Recent studies have revealed two main mechanisms for EV-mediated viral infection. First, EVs carry host proteins that make recipient cells more susceptible to SARS-CoV-2 infection. SARS-CoV-2 infection requires multiple steps including ACE2-mediated receptor-binding and TMPRSS2-mediated intracellular cleavage. Current studies identified ACE2 in EVs and demonstrated the transfer of ACE2 among various cell types via EVs [195]. This finding implies that SARS-CoV-2 may utilize a similar strategy to human immunodeficiency virus (HIV), regarding virus internalization, in which SARS-CoV-2 enters target cells via binding to exosomal ACE2. Similarly, we have shown that exosomes facilitate virus entry of adenovirus and HIV [196,197,198]. The second potential mechanism for EV-mediated viral entry involves one of the most abundantly expressed proteins on the surface of EVs, tetraspanin CD9 [199]. It is described that TMPRSS2 and CD9 work together in cleaving viral fusion glycoproteins and facilitate a quick entry coronavirus (e.g., MERS-CoV) into lung cells [200]. Furthermore, CD9 also accelerates lentiviral infection and enhances transduction efficiency in immune responsible cells including B cells and T lymphocytes [201]. These data reveal that CD9 and other tetraspanins on exosomal surface may be a mediator in SARS-CoV-2 infection.

During COVID-19 infections, especially in severely ill patients, hypoxemia, endothelial activation, and a profound inflammatory response are expected to increase expression of endothelial and leukocyte tissue factor (TF), which initiate coagulation cascade. Then, an active form of TF can be released, circulate throughout the entire body, and participate in sepsis-induced coagulopathy as well as thrombus formation. Importantly, such a TF is associated with large EVs [202]. Recently, SARS-CoV-2 infection is also reported to induce the release of TF-positive EVs into the circulation that are likely to be responsible for thrombosis in patients with COVID-19. The EV-TF activity was also correlated with severity and mortality [203].

According to a new hypothesis, SARS-CoV-2 could make its way into saliva through various ways, among which are via nasopharyngeal epithelium draining into the mouth, infected oral mucosal endothelial cells, secretion from infected salivary glands, blood plasma-crevicular fluid route to the oral cavity, and periodontal tissue exudate [204,205,206,207,208].The basis for the Rutgers saliva test can be traced to the ability of exosomes to transfer the human ACE2 receptor to recipient cells following fusion and internalization. This process takes place through ACE2 interaction with the S protein of SARS-CoV-2 prior to entrance into the cell, suggesting a role for exosomes in viral pathogenesis.

Mesenchymal stem cells (MSCs) have been found to show anti-inflammatory capabilities and regenerative properties, likely enabling them to work as an armory of treatments for COVID-19, such as cell-based therapy. Additionally, rather than cellular engraftment and response at the site, paracrine action via the release of EVs can be attributed to MSCs. The abovementioned two promising features of the MSCs warrants further research to figure out how beneficial and safe MSC-EVs will be. It will also be the crucial question to resolve: what will be the proposed role of MSCs with optimal use? Potential answers can be: (i) packages of natural immunomodulatory molecules; (ii) competitors to SARS-CoV-2; and/or (iii) as souped-up delivery vehicles carrying a suitable payload including small interfering RNAs (siRNAs), miRNAs or proteins [209]. As repurposing already available antiviral drugs with an improved composition can enhance the efficacy as well as safety profile of drug and in other point of relevance, EVs are thought to cause fewer systemic side effects than by any other vehicles available to us. EVs can be projected to provide targeted delivery of drug, such as protease inhibitors which are also projected to be the potential treatment regimens against SARS-CoV-2 [18,210]. Future research works are expected to unravel the potential implications of EVs in the expanding field of corona virology, especially on SARS-CoV-2.

## 14. Conclusions

Coronaviruses are a large group of viruses that cause disease in birds and mammals. The pathology of infection can range from mild symptoms ranging from the common cold to death (SARS, MERS). Many HCoVs have been identified to have emerged from bats. Despite our knowledge of HCoVs and coronaviruses found in companion animals, the world has been taken by storm by the arrival of SARS-CoV-2, which mediated the COVID-19 pandemic. In 2020, the world began a frantic race to produce effective non-toxic therapeutics as well as safe and effective vaccines. This was accomplished but still the world remains under siege. There is a continued need for basic science research in hopes of predicting and halting the emergence of new variants such as the Delta variant. The appearance of the Delta variant has now created a new “Pandemic of the un-vaccinated”. There is also a continued need for surveillance and for society to follow the guidance of the CDC, FDA, and WHO, and in doing so hopefully we can get back to “normal”.

## Figures and Tables

**Table 1:** Comparative features of seven HCoVs and associated diseases.

	HCoV-229E	HCoV-NL63	HCoV-HKU1	HCoV-OC43	MERS-CoV	SARS-CoV	SARS-CoV-2	Reference
Genus	Alpha-CoV	Alpha-CoV	Beta-CoV	Beta-CoV	Beta-CoV	Beta-CoV	Beta-CoV	[12]
Length (bp)	27,317	27,553	29,926	30,741	30,119	29,751	29,903	[12]
% Identity with SARS-Cov2	65.04	65.11	67.59	68.93	69.58	82.45	100	[12]
Accession No.	NC_002645	NC_005831	NC_006577	NC_006213	NC_015843	NC_004718	NC_045512	[12]
Case fatality rate (CFR) (%)	-*	-*	-*	-*	~35 (838/2428)	~10 (776/8000)	1–4 (4,526,583/218,205,951 as of 2 September 2021)	[43]
Basic Reproduction no. (R_0_)	-*	-*	-*	-*	>1	3	2–2.5	[43]
Host receptor	APN	ACE2	O-Acetylated Sialic Acid	O-Acetylated Sialic Acid	DPP4	ACE2	ACE2	[43,44,45,46]
Epidemiological effect	Endemic	Endemic	Endemic	Endemic	Regional outbreak	Global Pandemic	Global Pandemic	[12,45]

APN, Aminopeptidase N; DPP4, Dipeptidyl peptidase 4. Case fatality rate (CFR) is the percentage of the number of deaths out of the total number of cases. Basic reproduction number/ratio/rate (R_0_) of infection is the projected number of cases directly created by one case in a population where all individuals are equally prone to infection [47]. * As the HCoV-229E, HCoV-NL63, HCoV-OC43, and HCoV-HKU1 are endemic/community acquired HCoVs causing mild respiratory infections, not so much has been studied in epidemiological level [34], so CFR and R_0_ were not included for them.

## Data Availability

Data in this review article can be used or presented for research and analysis purpose by appropriately citing the article. Any enquiry can be made with either corresponding author at her email: qmatthews@alasu.edu. or first author at his email: hsharma1952@myasu.alasu.edu.
